# Al Matrix Composites Reinforced by Ti and C Dedicated to Work at Elevated Temperature

**DOI:** 10.3390/ma14113114

**Published:** 2021-06-06

**Authors:** Bartosz Hekner, Jerzy Myalski, Patryk Wrześniowski, Tomasz Maciąg

**Affiliations:** Faculty of Materials Engineering Krasińskiego 8, Silesian University of Technology, 40-019 Katowice, Poland; Jerzy.Myalski@polsl.pl (J.M.); Patryk.Wrzesniowski@polsl.pl (P.W.); Tomasz.Maciag@polsl.pl (T.M.)

**Keywords:** aluminium matrix composites, metal matrix composites, heterophase composites, Al-Ti-C system, carbon compounds

## Abstract

In this paper, the applicability of aluminium matrix composites to high-temperature working conditions (not exceeding the Al melting point) was evaluated. The behaviour of Al-Ti-C composites at elevated temperatures was described based on microstructural and phase composition observations for composites heated at temperatures of 540 and 600 °C over differing time intervals from 2 to 72 h. The materials investigated were aluminium matrix composites (AMC) reinforced with a spatial carbon (C) structure covered by a titanium (Ti) layer. This layer protected the carbon surface against contact with the aluminium during processing, protection which was maintained for the material’s lifetime and ensured the required phase compositions of Al_4_C_3_ phase limitation and AlTi_3_ phase creation. It was also proved that heat treatment influenced not only phase compositions but also the microstructure of the material, and, as a consequence, the properties of the composite.

## 1. Introduction

Aluminium matrix composites (AMCs) are among the most commonly used material solutions in many industries. Their applicability depends on the specific properties of the composite. When aluminium is used as a matrix, chemical resistance, thermal conductivity, and elasticity can be achieved in the material created. The low density of Al (2.7 g/cm^3^) leads to a reduction in composite mass compared to, e.g., Fe-based composites. However, the mechanical properties of pure aluminium are not sufficient for many of real working conditions. To achieve the required parameters, aluminium can be reinforced using a variety of ceramic materials, such as oxides (e.g., Al_2_O_3_) [1,2], carbides (e.g., SiC and WC) [2,3,4] or nitrides (e.g., Si_3_N_4_) [5,6]. These ceramic phases are commonly used to improve a material’s mechanical properties, e.g., hardness or stiffness [2,3,4,5,6]. Moreover, the applied reinforcement can also provide additional benefits. For example, carbon and its compounds are promising components for the improvement of AMC’s mechanical properties (e.g., carbon nanotubes [7] or graphene [8]) and also for tribological properties (e.g., graphite [9] or glassy carbon [10]). In highly developed materials solutions, additional reinforcements such as carbon are present around the main reinforcement phase (such as Al_2_O_3_ or SiC). These types of composites belong to the heterophase group of reinforced composites, where more than one compound affects the material’s properties [11,12,13].

The application of carbon in AMC is very limited due to the high reactivity between aluminium and carbon components. High-temperature processing leads to the creation of an aluminium carbide (Al_4_C_3_) phase, which is metastable in ambient conditions and strongly hydrophilic. This can result in a reduction in properties and, in extreme cases, destruction of the material. Moreover, this type of undesired phase can occur during the lifetime of AMC, which is especially dangerous for composites working at high temperatures under high load for long periods of time [14,15].

In this paper, innovative composites with heterophase reinforcements Ti-C and Al matrix were evaluated. The applied carbon consisted of an amorphous phase of carbon named glassy carbon, which was produced using our own method [16] in the shape of spatial, open-cell foam [17]. The titanium was in the form of a layer on the carbon surface used to prevent contact of C with melted Al during infiltration and subsequently through the material’s lifetime. Specific phases were used to increase the properties of the composite and also for their high potential for creating new phases between aluminium and titanium or titanium and carbon. The presence of these new phases led to improved mechanical properties and also provided high chemical bonding between phases, additionally increasing the properties of the created material.

**Figure 1:**
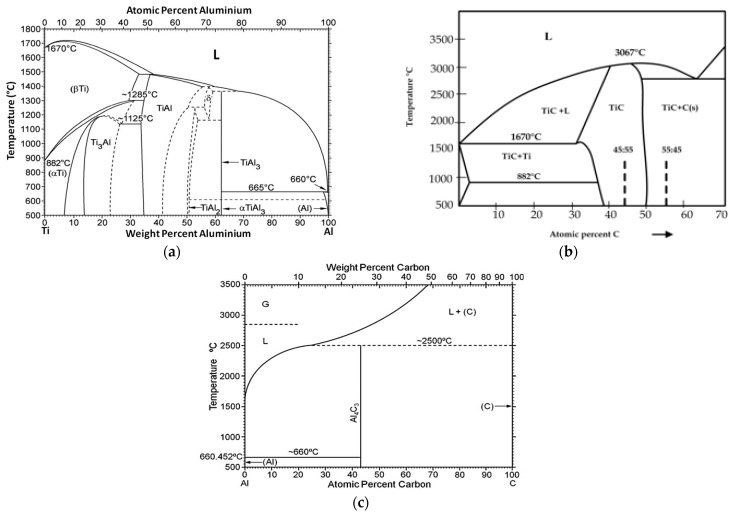
Phase diagram for (**a**) Ti-Al system [19]; (**b**) Ti-C system [22]; (**c**) Al-C system [23]; L—liquid, G—gas, C—carbon.

The potential for creation of new phases in an Al-Ti system was analysed based on the phase diagram for these compounds presented in Figure 1a [18,19]. According to this survey, the phase composition in Al-Ti strongly depends on component proportion. Al_3_Ti (tetragonal) is created when the minimum amount of Ti is about 24 mass.%. Increasing the amount of Ti leads to the creation of Al_2_Ti (orthorhombic), AlTi (tetragonal) and AlTi_3_ (hexagonal), respectively. The experimental investigation presented in [20] confirms the relationship between Ti-Al ratio and phase composition of the created materials. The presence of these intermediate phases increases material properties, such as melting point, chemical resistance and mechanical strength; however, these phases limit the material ductility and also increase fragility. 

Another area suitable for the creation of new phases is the border between Ti and C compounds. Analysis was based on the Ti-C phase diagram (Figure 1b) [21,22], which showed the Ti-C tendency to create titanium carbide phases in a whole range of compound content. However, the analysis of the crystal structure data showed that the aluminium carbide phase consisted of 32–48.8 at.% and 32–36 at.% for TiC and Ti_2_C, respectively [21], which approximates to 10–20 mass.% of carbon.

The phase diagram obtained at equilibrium conditions for an Al–C system revealed a tendency to create three types of phases, namely, aluminium, graphite and Al_4_C_3_, as the only intermediate compounds (Figure 1c) [23]. It is known that the aluminium carbide phase is metastable in ambient conditions, which limits the detectability of this phase in investigation conducted in stable conditions [24].

The supporting diagrams for the three-compounds system Al-Ti-C is presented in [25]. The authors took into account the phase composition of this system at 1000, 1100 and 1300 °C. This examination demonstrated the possibility of creating not only two-component phases (e.g., TiAl_2_ and Al_3_Ti) but also three-component phases, such as Ti_2_AlC. Moreover, the findings revealed a strong tendency to reaction of both components with melted aluminium. The isothermal sections of the Al-Ti-C system [25] showed that in a whole range of compounds, the new phases could be created at elevated temperatures.

The thermodynamic analyses presented in [26,27] and confirmed by experimental analyses [28] revealed a tendency to creation of inter-metallic phases in an Al-Ti-C system. A further thermodynamic analysis of an Al-Ti-C system [29] revealed that the most stable phase in this system is TiC. However, in some cases, this reaction is more complicated because additional, inter-metallic phases can be obtained. These results were also confirmed in experiments by the authors.

Based on the analysed diagrams and thermodynamic assumptions, it can be noted that component ratio influences the chemical composition of the analysed composite materials. However, high temperature is needed to obtain the expected new phases. In this paper, the possibility of creating new phases in an Al-Ti-C system was evaluated. To obtain the required reaction between phases, heat treatment over time was conducted. The parameters for the heat treatment investigations were comparable to actual working conditions, which provided an opportunity to predict material behaviour during its lifetime. 

## 2. Materials and Methods

**Figure 2:**
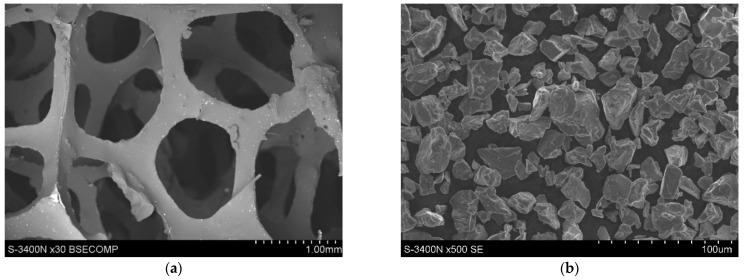
Microstructure of (**a**) spatial structure of PU foam; (**b**) Ti powder (SEM).

The evaluated materials were aluminium matrix composites (AMCs) reinforced by glassy carbon (GC) and titanium (Ti) phases. The glassy carbon with spatial structure was manufactured using our own production method [30]. The structure, similar to foam, was achieved by application of polyurethane (PU) foam as a precursor of shape (Figure 2a). This type of PU foam is widely used in industry, for example, as a filter for liquids. The main factor for the selection of foam was its structure—pores per inch (ppi)—and its impact on the glassy carbon amount and size of the single carbon area in the composite [31]. For evaluation, foam with a ppi of 45 was chosen as a result of own prior research.

In the next step, a thin layer of suspension of titanium particles in phenol–formaldehyde (P-F) resin was applied to PU foam walls via the immersion method. A high-purity titanium powder (>99%), with a particle size < 45 µm was applied. The microstructural view of Ti powder is presented in Figure 2b. A range of Ti particle suspensions were prepared (5, 10, 15, 20, 25, 30, 35 and 40 vol.%) in a relation to the resin (Table 1). All suspensions were cured adequately using a P-F resin curing process. Prepared 3D C-Ti structures were pyrolyzed at non-equilibrium conditions—1000 °C for 1 h in inert gas. Both polymer materials—PU and P-F resin—were carbonised to achieve glassy carbon—a pure carbon structure with a low level of additional components and an amorphous structure. The P-F resin, as a duroplastic material, prevents deformation of the PU foam during the pyrolysis process, which allowed for the 3D structure of the PU precursor to be preserved (Figure 2a).

**Figure 3:**
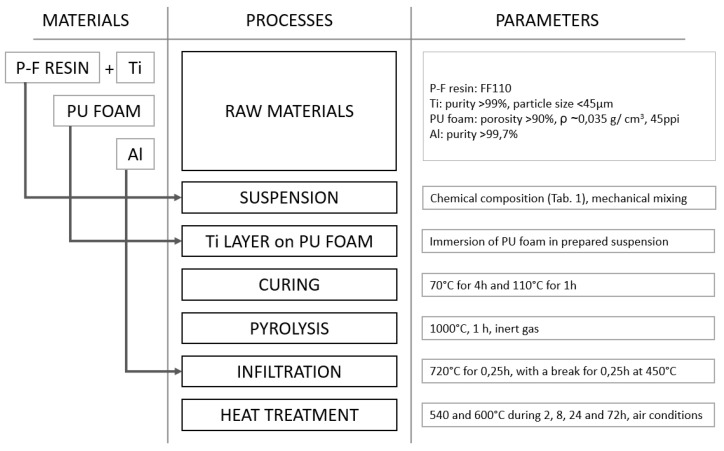
Schematic diagram of processes performed.

The prepared 3D C-Ti structures were infiltrated by high purity aluminium in a Degussa press at 720 °C for 15 min, with a break of 15 min at 450 °C. Under external pressure of 10 MPa under vacuum conditions, the optimum infiltration of spatial structures was achieved. The next part of our investigation was conducted to evaluate material behaviour in real-life, real-time conditions. During the heat treatment investigations, materials were heated for a range of times: 2, 8, 24, and 72 h, and at temperatures of 540 and 600 °C, under air conditions. The schematic diagram of processes performed is presented in Figure 3.

As part of the evaluation, the materials were inspected with scanning electron microscopy (SEM) (JEOL JCM-6000 Neoscope II, Tokyo, Japan and Hitachi S-3400, Krefeld, Germany) using the EDS technique. The thermogravimetric (TG) evaluation of Al-Ti-C composition was conducted in ambient gas at temperatures up to 1200 °C. All components were mechanically mixed in an initial powder form. The powders consisted of equal proportions of each component (Al-Ti-C). The phase compositions were determined using a PANalytical Empyrean X-Ray Diffractometer (Almelo, Netherlands) for the powder form and XRD X’Pert 3 (Almelo, Netherlands) for solid materials. Mechanical properties were described based on hardness measurement using the Vickers method HV 0.2.

## 3. Results

**Figure 4:**
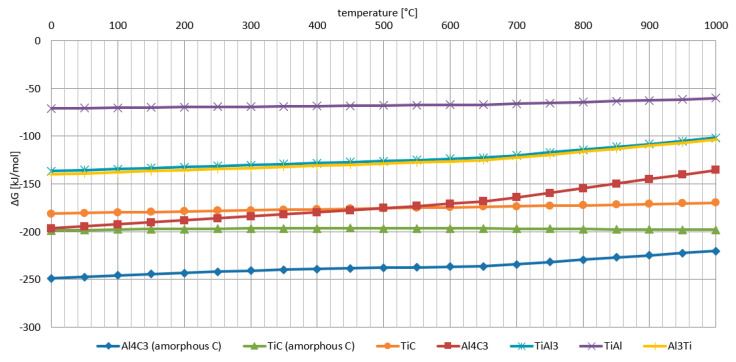
Free energy of formation in an Al-Ti-C (amorphous) system.

An initial material evaluation using thermodynamic analysis was performed for all components used in composite creation (Figure 4). This analysis revealed that the most likely phase creations were AlTi, TiAl_3_ and Al_3_Ti. The free energy of formation was lower for phases with an amorphous structure than those with a crystal structure. 

**Figure 5:**
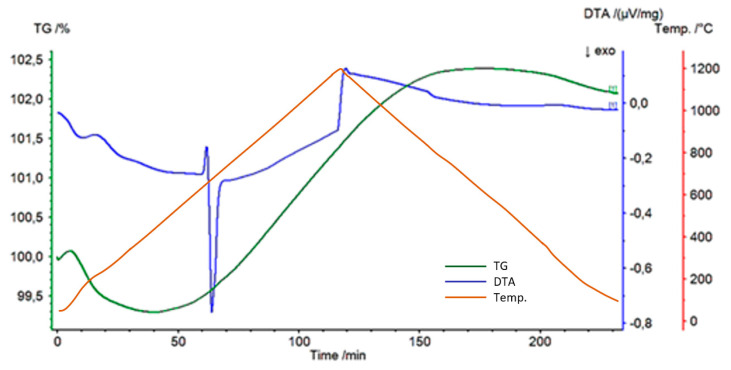
Thermogravimetric analysis of an Al-Ti-C system (TG).

In the next step, the prepared composition of Al-Ti-C was evaluated in a powdered, initial (non-processed) form in a TG test. Based on the results presented in Figure 5, the potential reaction in the analysed system was determined. A temperature of 1200 °C was applied, which is higher than the processing temperature of carbon, to determine whether any additional components (non-pyrolysed) appeared in these conditions. Under the influence of temperature, two reactions appeared: firstly an endothermic reaction at about 670 °C; secondly an exothermic reaction, which manifested immediately after the first one at about 700 °C. A mass changes analysis revealed a decrease of about 0.8% at the start of heating, followed by an increase of about 3%.

**Figure 6:**
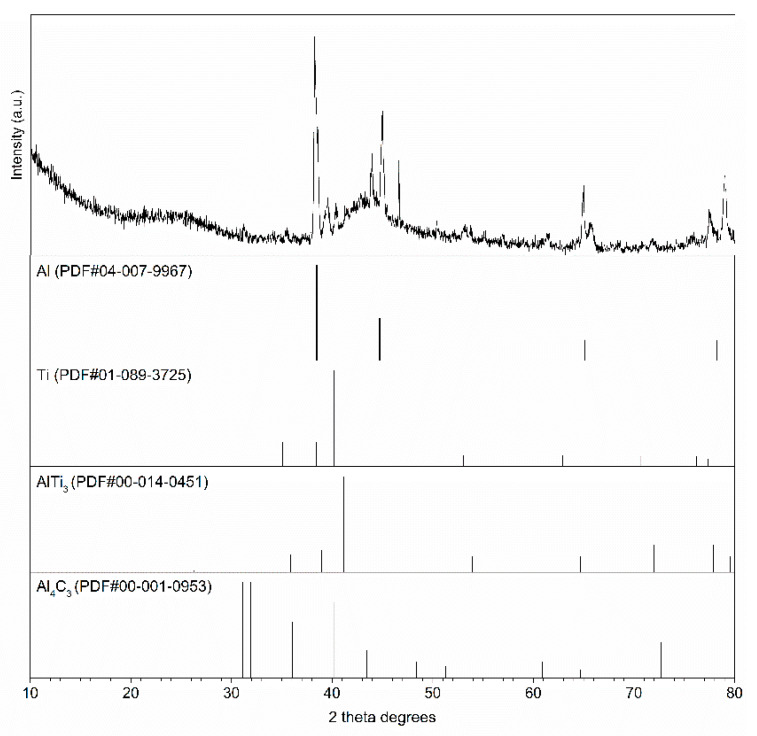
Phase composition of Al-Ti-C system after thermogravimetric analyses at 1200 °C (XRD).

For material after TG investigation, phase analyses were conducted to determine new phases created in the Al-Ti-C system at a temperature of 1200 °C. Figure 6 presents the result of the examination. A reaction between the aluminium and titanium led to AlTi_3_ phase creation, whilst the aluminium–carbon system revealed a tendency to creation of a metastable Al_4_C_3_ phase. There was no reaction between the titanium and carbon components.

**Figure 7:**
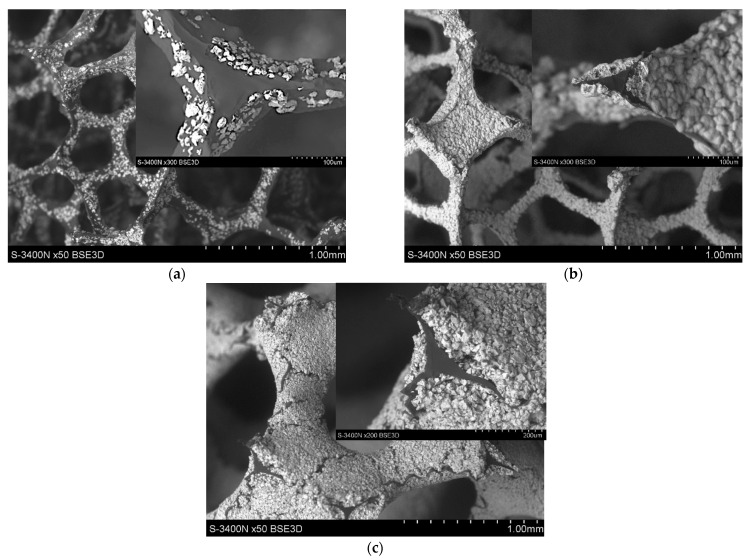
The microstructure of the obtained reinforcement layers varied according to Ti particle suspension: (**a**) 10 vol.%; (**b**) 25 vol.%; (**c**) 40 vol.% in PF resin suspension. In insets, the cross-sections of foam are presented (SEM).

The microstructure observations of the created spatial forms of heterophase reinforcements revealed the influence of the amount of titanium on the quality of the created layer on GC foam. With lower volumes of Ti particle suspension, the created layer was discontinuous and did not cover the whole carbon surface. This phenomenon is presented in Figure 7a and its inset. Increasing the volume of Ti particle suspension led to improvements in the continuity of the Ti layer. The expected microstructure was achieved with a suspension of 25 vol.% of titanium particles in P-F resin. The created layer fully covered the carbon surface, as shown in Figure 7b. The thickness of the Ti layer was approximately the size of a single Ti particle (Figure 7b inset). With reinforcements with a Ti particle suspension volume higher than 25 vol.%, undesired microstructural aspects were noted. On the surface of the created layer, some discontinuity was noted (Figure 7c). However, the microstructural defects relate to a high volume of brittle particles. The size of the created layer was more than 100µm, which was greater than the Ti particle size (Figure 7c inset). Moreover, at the processing temperatures applied (1000 and 720 °C for pyrolysis and the infiltration process, respectively), the titanium particles were not melted (melting point of Ti: 1668 °C). In the created layer, adhesion bonding of Ti was supported by the carbon bonding obtained as a result of the P-F resin carbonisation process. 

**Figure 8:**
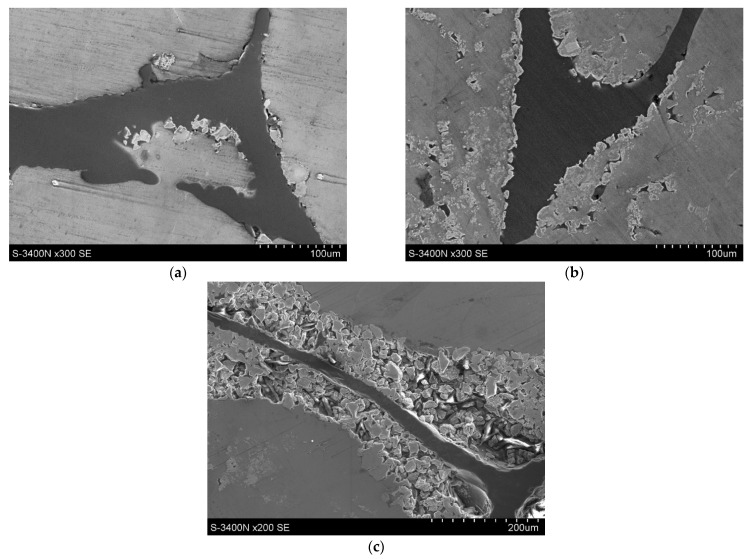
The microstructure of composites obtained by infiltration of spatial structures with a range of Ti particle suspensions: (**a**) 5 vol.%; (**b**) 20 vol.%; (**c**) 40 vol.% used for Ti layer creation (SEM).

**Figure 9:**
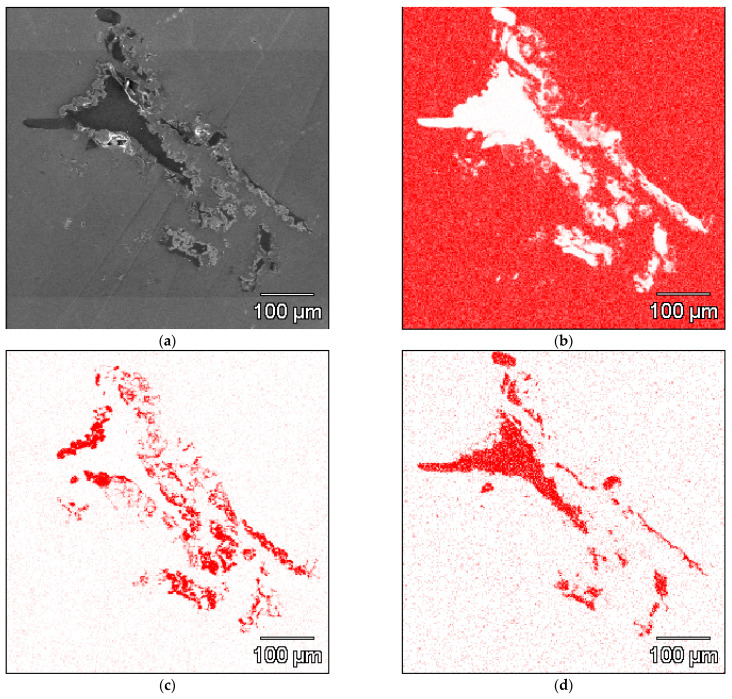
(**a**) The microstructure and chemical distribution of (**b**) aluminium; (**c**) titanium; (**d**) carbon compounds in composite with layers created by a suspension of 20 vol.% of Ti (SEM + EDS).

The analysis of the microstructures of the composites after infiltration by aluminium (Figure 8) confirmed observations for Ti-C spatial reinforcement. The quality of the composite depends on the amount of titanium particles in suspension. With low Ti particle suspension, the discontinuity in the Ti layer led to direct contact of melted aluminium with the carbon reinforcement. This phenomenon is presented in Figure 8a. For composites with the highest Ti particle suspension (more than 30 vol.%), the created layer revealed high porosity due to poor bonding with unmelted Ti particles. In material with 40 vol.% of Ti particles in suspension, the size of the created layer was in the range of 50–200 µm. The optimal areas for void creations were Ti areas with high width, which can be seen in Figure 8c. The required quality of the microstructure was achieved for materials with Ti particle suspensions in a range of 20–30 vol.%. The titanium particles created a consistent layer on the carbon surface. The microstructure is shown in Figure 8b. Moreover, the presence of loose Ti particles (not bonded to spatial structure) was observed (Figure 9c). These particles are probably a result of turbulent liquid aluminium flow during the infiltration process. The distribution of reinforcement phases presented in Figure 9 confirms the required spatial structure of reinforcement (shape of foam) but also reveals that the area of reinforcement present is higher than the theoretical assumption (loose particles of Ti and C in Al matrix).

**Figure 10:**
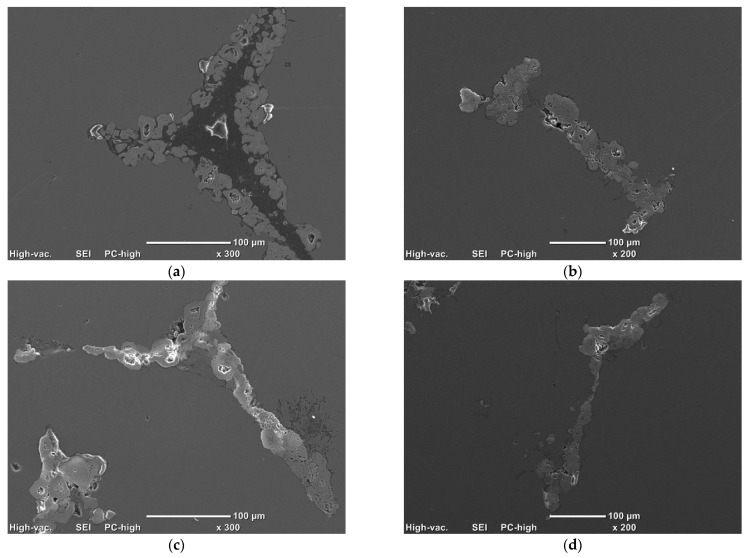
The microstructures of composites after heat treatment at 600 °C at varying times: (**a**) 2 h; (**b**) 8 h; (**c**) 24 h; (**d**) 72 h (SEM).

Based on the knowledge gained on microstructural aspects and their influence on the final quality of composite material, AMC with a 20 vol.% of Ti was selected for heat treatment investigations. The applied temperatures, 540 and 600 °C, were adequate for 0.8 and 0.9 of the aluminium melting point, respectively. Under heating conditions, the structure of the analysed composites changed, especially in the carbon areas, as shown in Figure 10. The main influence on the reinforcement structure was the duration of processing. At the start of the heating process (2 h), the characteristic geometric shape of glassy carbon foam was found in some areas, dependent on C width. The larger size of carbon reinforcement affected the presence of pure carbon in the microstructure (Figure 10a). After a period of time at high temperature (8–72 h), the Ti-C areas did not reveal a clear boundary between phases in comparison to the initial stage. Moreover, the heat treatment led to increased porosity. The optimal areas for void creation were those with sufficient carbon presence and the boundaries between titanium reinforcement and aluminium matrix. Similar microstructural changes were seen at both 540 and 600 °C.

**Figure 11:**
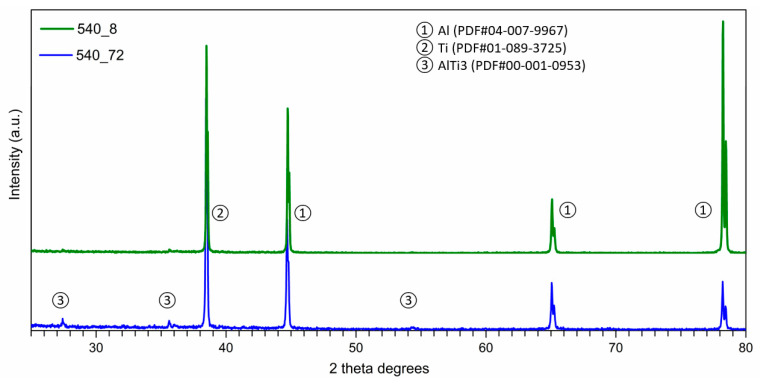
Phase composition for AMC-Ti-C composite after heat treatment at 540 °C, over 8 and 72 h durations (XRD).

The microstructural changes observed after heating were evaluated by phase analysis in the areas of composite reinforcement. The result of the XRD examination is shown in Figure 11. Firstly, an absence of the aluminium carbide phase (Al_4_C_3_) was observed, even after 72 h of heating at 600 °C. The composites at the start of heat treatment (>8 h) revealed the same phase composition as material without additional processing, which further revealed a low tendency to creation of new phases in Al-Ti-C systems in these conditions. After more prolonged heating (≥24 h), new phases were created. The creation of AlTi_3_ was noted for composites heated at both 540 and 600 °C.

**Figure 12:**
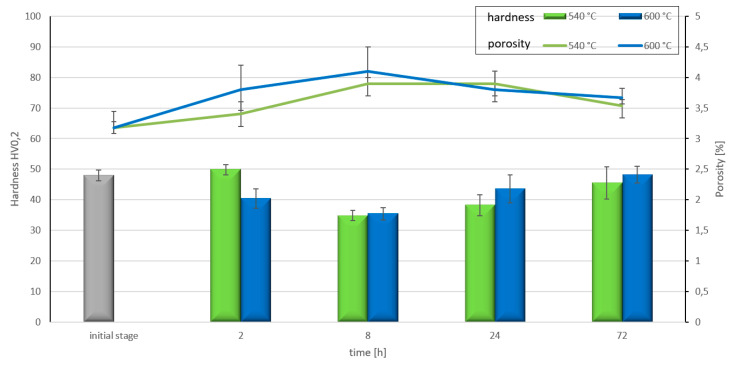
Hardness and porosity of Al-Ti-C composites after heat treatment at different times (2–72 h) and temperatures (540 and 600 °C).

The changes in material properties were described based on the hardness measurement in areas of the aluminium matrix. Hardness results are presented in Figure 12. The significant influence of heat treatment on hardness was noted. After short periods of time (2 h at 600 °C and 8 h at 540 °C), a decrease in hardness was noted. Over longer periods (>24 h) at both temperatures, the hardness increased up to a level similar to the hardness of the matrix in composites without heat treatment.

## 4. Discussion

The initial step of the examination was focused on the reactivity of an Al-Ti-C system. Based on knowledge gained via TG testing and subsequent XRD analysis, it was noted that the first chemical reaction appeared at temperatures higher than the melting point of aluminium (Figure 5). The exothermic reaction noted at about 700 °C relates to an Al-Ti system and corresponds to AlTi_3_ phase creation (Figure 6). A mechanism of new phase creation was presented in [32]. The influence of new phases on mechanical properties as a result of precipitation hardening and alloying mechanisms is presented in [33,34,35,36]. However, XRD investigation revealed a tendency to undesired reactions between aluminium and carbon after heating to 1200 °C (Figure 6). A first finding was that a limitation in the contact area between aluminium and carbon is needed in order to control the phase composition in the material.

In the literature, many manufacturing solutions for aluminium matrix composites reinforced by carbon are described [33,34,35,36,37]. However, no solution for avoiding contact between carbon and aluminium has been presented. Other authors [32,33,37] present methods for obtaining phase composition through the control of the chemical compositions of material, but this solution limits the C component content in the finished material. This does not support composite design, e.g., for tribological properties, which strongly depend on carbon content in Al matrix composites [38,39].

In presenting this paper, an innovative spatial form of reinforcement was proposed in order to control the phase composition during processing and the subsequent lifetime of the material (Figure 2a). The reinforcement comprised glassy carbon foam covered by titanium particles (Figure 7). The limitation of contact between C and the Al matrix was achieved through selection of the optimum Ti amount (Table 1, Figure 7b). Based on our investigations, it can be noted that the Ti layer should be equal to the Ti particle size. This was achieved for composites with a 20 vol.% of Ti in suspension. This solution gave an opportunity to produce Al matrix composites with high C content without creating unwanted phases in the Al-C system.

The composites with spatial Al-Ti-C structure were heat treated at a temperature equal to between 0.8 and 0.9 of the Al melting point. These parameters correspond to normal working conditions. This part of the investigation proved that Al-Ti phases seen at temperatures of about 700 °C (Figure 5 and Figure 6) can also be created at lower temperatures, over a longer time (Figure 11). Another achievement was the avoidance of theAl_4_C_3_ phase even with high-temperature processing (0.9 melting point of aluminium) and a long period of heat treatment (72 h). The presence of the Ti layer prevented unwanted reactions in the Al-C system but supported the Al-Ti system reaction (Figure 11).

The treatment we carried out also influenced the microstructure and properties of the material. The changes in GC at the start of heat treatment led to higher porosity and decreased hardness (8 h) (Figure 10 and Figure 12). A longer heating time (24 h) resulted in a new AlTi_3_ phase creation and improvement in the mechanical properties as a consequence (Figure 12). The observed alloying mechanisms are described in many publications. Other authors [40,41] describe the influence of Ti and the amount of compound used on the mechanical properties of Al matrix composites. A further investigation showed the strong influence of manufacturing technique on the composite properties [42]. The hardness achieved corresponds to results achieved with Al matrix composites reinforced by Ti or its compound, obtained by conventional methods, as documented in the literature. Additionally, in this study, it was found that after longer heating times, the boundaries between C and Ti phases were unclear and the porosity decreased (Figure 10 and Figure 12). These microstructural changes also correspond to the increase in material properties (Figure 12). The changes in phase composition and microstructure proved that the material created can be successfully applied in elevated temperatures. 

Based on these results, it can be seen that Al matrix composites with spatial C-Ti reinforcement can be successfully applied at high temperature and/or in high-load application (e.g., friction points), which will provide a basis for the authors’ next investigation.

## 5. Conclusions

For the manufacturing of AMC reinforced by Ti and C, the following should be taken into account:

- The Al-Ti-C system revealed a tendency for the creation of new phases, especially in Al-Ti and Al-C systems. AlTi_3_ and Al_4_C_3_ phases were detected after TG investigation in the evaluated composites.
- To control the phase composition of the material, a thin layer of Ti particles was applied on the surface of a C spatial structure. This solution limits contact between Al and C compounds and avoids the presence of an unwanted Al_4_C_3_ phase as a result.
- Using titanium as a material for the creation of a protective layer, reactions (AlTi_3_) with the aluminium matrix, which benefit the material’s properties, are possible.
- The optimal quality of protective Ti layer (tightness, continuity) can be achieved by adjustment of the compound’s ratio. The desired structure was achieved with reinforcement foam covered by suspension of 20 vol.% of Ti in PA resin.
- The optimum conditions for new phase creation in Al-Ti systems are high temperature (higher than 0.8 of the melting point of aluminium) and prolonged treatment duration (minimum 24 hours). With these parameters, the behaviour of Al-Ti-C composites in real-time working conditions (high temperature and load) can be better understood.

## Figures and Tables

**Table 1:** Chemical compositions of suspension used to produce spatial Ti-C reinforcement.

Volume Ratio (%)	Ti	5	10	15	20	25	30	40
	P-F resin	95	90	85	80	75	70	60
mass (g)	Ti	8.98	17.96	26.94	35.92	44.90	53.88	71.84
	P-F resin	45.60	43.20	40.80	38.40	36.00	33.60	28.80

## Data Availability

Data is contained within the article.
